# A class II MHC-targeted vaccine elicits immunity against SARS-CoV-2 and its variants

**DOI:** 10.1073/pnas.2116147118

**Published:** 2021-10-15

**Authors:** Novalia Pishesha, Thibault J. Harmand, Paul W. Rothlauf, Patrique Praest, Ryan K. Alexander, Renate van den Doel, Mariel J. Liebeskind, Maria A. Vakaki, Nicholas McCaul, Charlotte Wijne, Elisha Verhaar, William Pinney, Hailey Heston, Louis-Marie Bloyet, Marjorie Cornejo Pontelli, Ma. Xenia G. Ilagan, Robert Jan Lebbink, William J. Buchser, Emmanuel J. H. J. Wiertz, Sean P. J. Whelan, Hidde L. Ploegh

**Affiliations:** ^a^Society of Fellows, Harvard University, Cambridge, MA 02138;; ^b^Program in Cellular and Molecular Medicine, Boston Children's Hospital, Harvard Medical School, Boston, MA 02115;; ^c^Klarman Cell Observatory, Broad Institute of MIT and Harvard, Cambridge, MA 02142;; ^d^Department of Immunology and Infectious Diseases, Harvard School of Public Health, Boston, MA 02115;; ^e^Department of Molecular Microbiology, Washington University School of Medicine, St. Louis, MO 63110;; ^f^Program in Virology, Harvard Medical School, Boston, MA 02115;; ^g^Department of Medical Microbiology, University Medical Center Utrecht, 3584 CX Utrecht, The Netherlands;; ^h^Department of Genetics, Washington University School of Medicine, St. Louis, MO 63110;; ^i^High Throughput Screening Center, Washington University School of Medicine, St. Louis, MO 63110;; ^j^Department of Biochemistry and Molecular Biophysics, Washington University School of Medicine, St. Louis, MO 63110

**Keywords:** nanobody, COVID-19, vaccine

## Abstract

The pandemic caused by severe acute respiratory syndrome coronavirus 2 (SARS-CoV-2) has resulted in over 100 million infections and millions of deaths. Effective vaccines remain the best hope of curtailing SARS-CoV-2 transmission, morbidity, and mortality. The vaccines in current use require cold storage and sophisticated manufacturing capacity, which complicates their distribution, especially in less developed countries. We report the development of a candidate SARS-CoV-2 vaccine that is purely protein based and directly targets antigen-presenting cells. It consists of the SARS-CoV-2 Spike receptor-binding domain (Spike_RBD_) fused to an alpaca-derived nanobody that recognizes class II major histocompatibility complex antigens (VHH_MHCII_). This vaccine elicits robust humoral and cellular immunity against SARS-CoV-2 and its variants. Both young and aged mice immunized with two doses of VHH_MHCII_-Spike_RBD_ elicit high-titer binding and neutralizing antibodies. Immunization also induces strong cellular immunity, including a robust CD8 T cell response. VHH_MHCII_-Spike_RBD_ is stable for at least 7 d at room temperature and can be lyophilized without loss of efficacy.

## Significance

Vaccines remain the best hope of curtailing SARS-CoV-2 transmission, morbidity, and mortality. Currently available vaccines require cold storage and sophisticated manufacturing capacity, complicating their distribution, especially in less developed countries. We report a protein-based SARS-CoV-2 vaccine that directly and specifically targets antigen-presenting cells. It consists of the SARS-CoV-2 Spike receptor-binding domain (Spike_RBD_) fused to a nanobody that recognizes class II major histocompatibility complex antigens (VHH_MHCII_). Our vaccine elicits robust humoral (high-titer binding and neutralizing antibodies) and cellular immunity against SARS-CoV-2 and its variants in both young and aged mice. VHH_MHCII_-Spike_RBD_ is stable for at least 7 d at room temperature and can be lyophilized without loss of efficacy, desirable attributes for logistical reasons.

Severe acute respiratory syndrome coronavirus 2 (SARS-CoV-2), the causative agent of COVID-19, has caused a global pandemic, infecting over 230 million people, and leading to millions of deaths (1). Rapid distribution of effective vaccines on a global scale is the most effective means of mitigating the political, social, and economic destabilization caused by the SARS-CoV-2 pandemic.

The SARS-CoV-2 spike (S) protein is a trimeric transmembrane protein that binds to the cell surface receptor angiotensin-converting enzyme 2 (ACE2) via its receptor-binding domain (RBD) and mediates fusion with host membranes (2). SARS-CoV-2 S is the primary target for neutralizing antibodies and elicits both CD4 and CD8 T cell responses during infection (3–7). Most vaccines in current use or in development target S, or fragments of S, as the primary antigen (8). Because several variants of concern have emerged, many of which contain mutations in S that partially resist neutralization by vaccine-elicited and COVID-19–elicited antibodies, vaccines that offer protection against new variants are necessary (9–11).

Leading vaccine candidates use an array of diverse vaccine platforms. These include inactivated virions, DNA-based vaccines, recombinant subunit preparations, lipid-encapsulated mRNA formulations, as well as live-attenuated, replication-incompetent viral vectored, and replication-competent viral vectored vaccines (8). None of them directly and specifically target antigen-presenting cells (APCs). We hypothesized that targeted delivery of antigen to professional class II MHC^+^ APCs would improve access to the processing and presentation pathways that generate CD4 and CD8 T cell responses, in addition to provoking a robust antibody response. Our earlier efforts to generate an anti-HPV16 CD8 T cell response relied on fusions of an anti-CD11b nanobody to the immunodominant epitope of the HPV16 E7 protein as a vaccine. Its success in eradicating even established tumors inspired us to pursue a similar effort to deliver the RBD of the SARS-CoV2 S protein as a fusion with a nanobody that targets APCs (12). Most vaccines in current use require specialized storage conditions (13–15). The development of vaccines with enhanced stability to allow storage at ambient temperature and rapidly adjustable to emerging variants of the virus therefore remains a priority. Moreover, vaccines that can be produced rapidly in a scalable manufacturing process would improve access.

Here we report the development of a recombinant protein vaccine that consists of the SARS-CoV-2 Spike RBD (Spike_RBD_) fused to an alpaca-derived nanobody that targets class II major histocompatibility (MHC II) complex antigens (VHH_MHCII_-Spike_RBD_). This vaccine delivers the antigen directly to class II MHC^+^ APCs. Immunization of both young and aged mice with two doses of VHH_MHCII_-Spike_RBD_ resulted in robust binding and neutralizing antibody responses against SARS-CoV-2 and emerging variants. Immunization also induced prominent CD8 T cell responses against conserved Spike_RBD_-derived epitopes. VHH_MHCII_-Spike_RBD_ can be produced in high yield in mammalian cells and tolerates both storage at room temperature for at least 7 d and lyophilization without loss of efficacy.

## Results

### VHH_MHCII_-Spike_RBD_ Elicits High-Titer Anti-Spike_RBD_ and Neutralizing Antibodies in Mice.

**Fig. 1.**
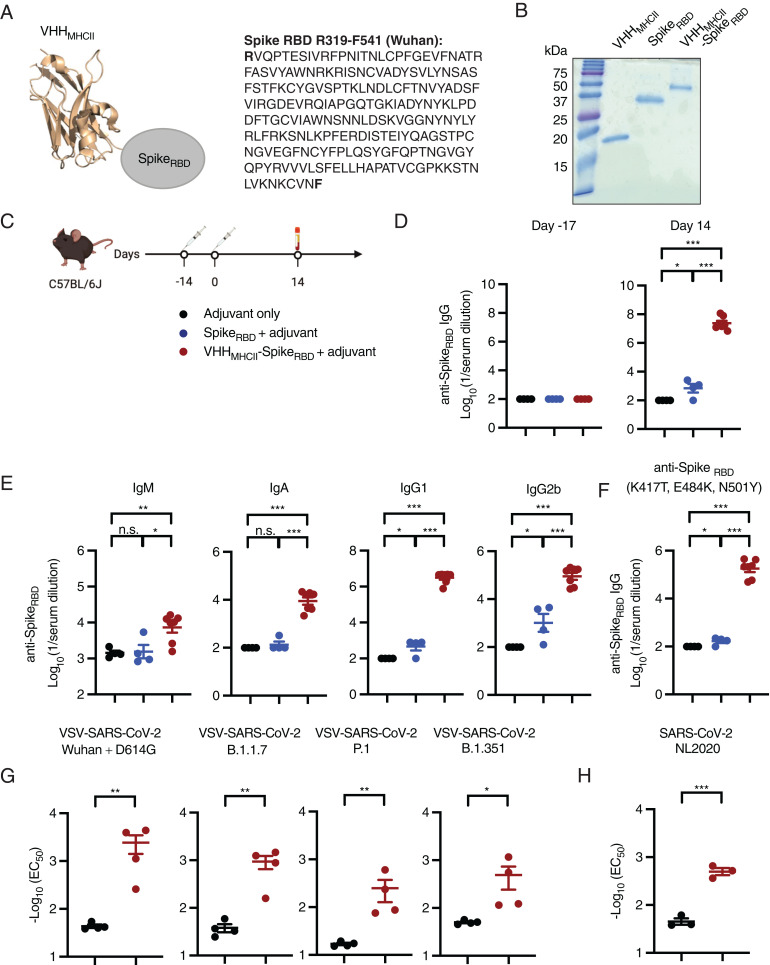
Immunization with VHH_MHCII_-Spike_RBD_ induces high-titer anti-Spike_RBD_ and neutralizing antibodies in mice. (*A*) Schematic of VHH_MHCII_-Spike_RBD_. The structure shown is a representative example of a VHH. (*B*) Coomassie-stained SDS/PAGE gel of purified VHH_MHCII_, Spike_RBD_, and VHH_MHCII_-Spike_RBD_. (*C*) C57BL/6J mice were immunized i.p. with adjuvant only, adjuvanted Spike_RBD_, or adjuvanted VHH_MHCII_-Spike_RBD_ on the indicated days. Serum samples were collected as indicated. (*D* and *E*) Total IgG (day −13 and day 14), or IgM, IgA, IgG1, and IgG2b (day 14) responses were evaluated from sera of immunized mice (*n* = 4 to 7 per group) by ELISA against recombinant Spike_RBD_. ELISA data were summarized as endpoint titers and presented as means ± SEM. (*F*) Humoral responses in sera of immunized mice were evaluated (*n* = 4 to 7 per group) by ELISA for anti-Spike_RBD_ (K417T, E484K, N501Y mutations) IgG. ELISA data were summarized as endpoint titers and presented as means ± SEM. (*G*) Neutralization data for VSV, pseudotyped with the SARS-CoV-2 Spike glycoprotein Wuhan + D418G and other indicated variants. (*H*) Neutralization assay against clinical isolates of SARS-CoV-2/NL/2020 strain. All data are presented as means ± SEM; n.s., not significant; *P < 0.05, **P < 0.01, ***P < 0.001, unpaired *t* test with Holm–Sidak adjustment.

We have characterized a single-domain antibody fragment that binds class II MHC antigens (VHH_MHCII_) with nanomolar affinity. Immunization of mice with an influenza A virus (IAV) HA2 antigen, conjugated to VHH_MHCII_, protected mice against a lethal IAV challenge (16). To apply this vaccine platform to SARS-CoV-2, we generated a recombinant protein that consists of a fusion between VHH_MHCII_ and the SARS-CoV-2 RBD (Fig. 1*A*). VHH_MHCII_-Spike_RBD_ was expressed in Expi293 cells and purified by means of its C-terminal His_6_-tag followed by size-exclusion chromatography. The identity of the product was confirmed by sodium dodecyl sulfate polyacrylamide gel electrophoresis (SDS/PAGE) (Fig. 1*B*). A 200-mL culture of Expi293 cell supernatant yielded ∼20 mg of recombinant protein.

To examine the immunogenicity of VHH_MHCII_-Spike_RBD_, we used a two-dose immunization regimen. Preimmune serum was collected at −17 d and C57BL/6J mice (H-2^b^ haplotype) were primed (day −14) intraperitoneally (i.p.) with adjuvanted (poly dIdC and anti-CD40 monoclonal antibody) VHH_MHCII_-Spike_RBD_ (20 μg), with an equimolar amount of adjuvanted Spike_RBD_ (13.5 μg), or with adjuvant alone. Mice were boosted with each corresponding preparation 14 d postprime (day 0) (Fig. 1*C* and *SI Appendix*, Fig. S1*A*). Serum was collected from all animals 14 d postboost (day 14). IgG titers were determined by ELISA against recombinant SARS-CoV-2 Spike_RBD_ (Wuhan Hu-1 strain) (Fig. 1*D* and *SI Appendix*, Fig. S1*B*). Immunization with two doses of VHH_MHCII_-Spike_RBD_ elicited high levels of anti-Spike_RBD_ antibodies in all animals, reaching mean endpoint titers in excess of 1/23,600,000, an approximate 34,000-fold increase over mice immunized with two doses of Spike_RBD_. Analysis of immunoglobulin subclasses showed evidence of class switching in mice immunized with VHH_MHCII_-Spike_RBD_, judged by the levels of IgA, IgG1, and IgG2b detected at day 14 (Fig. 1*E* and *SI Appendix*, Fig. S1*C*). Mean endpoint titers were higher in mice immunized with VHH_MHCII_-Spike_RBD_ than in mice immunized with Spike_RBD,_ with mean endpoint titers reaching >1/7,300 (IgM; fourfold increase over Spike_RBD_), 1/8,900 (IgA; 66-fold increase over Spike_RBD_), 1/3,160,000 (IgG1; 6,870-fold increase over Spike_RBD_), and ∼1/90,000 (IgG2b; 87-fold increase over Spike_RBD_). Immunization with VHH_MHCII_-Spike_RBD_ induced high levels of IgA, which suggests improved mucosal protection in immunized animals. Immunization of mice with two doses of VHH_MHCII_-Spike_RBD_ thus induces a robust humoral immune response, significantly higher than that induced by the Spike_RBD_ alone.

Since VHH_MHCII_-Spike_RBD_ binds class II MHC antigens encoded by the I-A locus on target APCs, we immunized a different inbred mouse strain (BALB/cJ; H-2^d^ haplotype) to confirm efficacy across different MHC haplotypes. Immunization with two doses of VHH_MHCII_-Spike_RBD_ led to comparable levels of total IgG in C57BL/6J (H-2^b^) and BALB/cJ (H-2^d^) mice (*SI Appendix*, Fig. S2 *A* and *B*). Immunoglobulin class switching was evident in both mouse strains, and mean Spike_RBD_-specific IgM, IgA, IgG1, and IgG2b titers were comparable (*SI Appendix*, Fig. S2*C*).

We next measured total and subclass immunoglobulin levels at days 7, 14, and 21 in mice immunized with one or two doses of adjuvanted VHH_MHCII_-Spike_RBD_ or control (*SI Appendix*, Fig. S3*A*). Mice immunized with two doses of VHH_MHCII_-Spike_RBD_ quickly achieved peak IgG titers on day 7, with levels persisting until at least day 21 (*SI Appendix*, Fig. S3*A*), while animals immunized with a single dose produced lower levels of total IgG and other subclass immunoglobulins (*SI Appendix*, Fig. S3*B*).

Because SARS-CoV-2 variants with mutations in S that enable partial immune escape have emerged, we next examined whether serum from mice immunized with VHH_MHCII_-Spike_RBD_ recognized recombinant Spike_RBD_ with the K417T, E484K, and N501Y mutations. These mutations are found individually in many S variants and in combination in the P.1 (Gamma or Brazil) variant and partially in the B.1.351 (Beta or South Africa) variant which has K417N instead of K417T (17). Immunization with two doses of adjuvanted wild-type Spike_RBD_ induced antibodies (day 14) with a low capacity to bind the triple-mutant Spike_RBD_, while immunization with two doses of adjuvanted VHH_MHCII_-Spike_RBD_ elicited antibodies still capable of recognizing the mutant Spike_RBD_ to high titers (mean endpoint titer of 1/178,000) (Fig. 1*F* and *SI Appendix*, Fig. S1*D*). Two doses of VHH_MHCII_-Spike_RBD_ were required to maintain high titers of IgG against the mutant Spike_RBD_ (*SI Appendix*, Fig. S3*C*).

We next measured the levels of neutralizing antibodies induced by immunization with two doses of adjuvanted VHH_MHCII_-Spike_RBD_ by using replication-competent vesicular stomatitis viruses that express eGFP and variants of the SARS-CoV-2 spike (VSV-SARS-CoV-2) in place of their native glycoprotein (18, 19). Mice immunized with two doses of adjuvant failed to neutralize VSV-SARS-CoV-2 expressing the Wuhan Hu-1+D614G spike, as well as those carrying the spikes of B.1.1.7 (Alpha or United Kingdom), P.1, and B.1.351 variants (Fig. 1*G*) (20). Alternatively, immunization with two doses of adjuvanted VHH_MHCII_-Spike_RBD_ induced high-titer neutralizing antibodies against VSV-SARS-CoV-2 expressing the spikes of Wuhan Hu-1+D614G (mean effective concentration, 50% [EC_50_] titers of 1/2,426), B.1.1.7 (1/937), P.1 (1/250), and B.1.351 (1/488). Sera from mice immunized with two doses of VHH_MHCII_-Spike_RBD_ neutralized a clinical isolate of SARS-CoV-2 strain NL/2020 (mean EC_50_ titers of 1/499), as measured by a quantitative RT-PCR assay (*SI Appendix*, Fig. S4*A*). Neutralizing titers were comparable to that of a monoclonal neutralizing antibody, 47D11 (1/640 to 1/1280 dilution range, 0.05 μg/mL to 0.025 μg/mL) (Fig. 1*H* and *SI Appendix*, Fig. S4*B*) (21). While neutralizing titers were impacted by mutations in the RBD of circulating variants, mean EC_50_ titers remain high. Regardless, high neutralizing titers against the Wuhan Hu-1+D614G after immunization with a matched RBD suggests that VHH_MHCII_-Spike_RBD_ carrying mutations found in the RBDs of circulating variants will significantly improve neutralizing titers against those variants. Other variants remain to be tested.

### A Single Dose of VHH_MHCII_-Spike_RBD_ Elicits Strong Cellular Immunity.

**Fig. 2.**
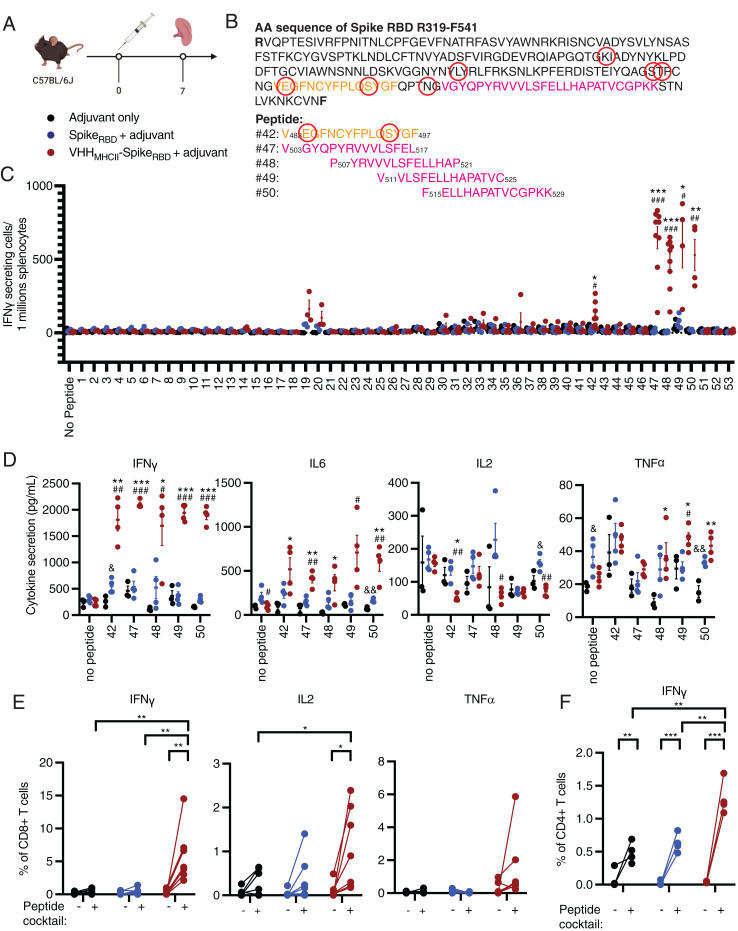
Immunization of mice with a single dose of VHH_MHCII_-Spike_RBD_ elicits strong cellular immunity. (*A*) C57BL/6J mice were immunized i.p. with one dose of adjuvanted VHH_MHCII_-Spike_RBD_. Spleens were harvested 7 d postimmunization. (*B*) Amino acid sequence of the SARS-CoV-2 Spike_RBD_. Residues circled in red denote residues in the Spike_RBD_ that are frequently mutated in circulating variants, including L452, S477, T478, E484, S494, and N501. Orange residues indicate a T cell stimulatory region of Spike_RBD_, while magenta residues indicate a second T cell stimulatory peptide region of Spike_RBD_. (*C*) The number of IFNγ-secreting cells in immunized mice was evaluated by ELISpot assays. Numbers on the *x* axis correspond to specific 15-mer peptides with 11-residue overlaps in the Spike_RBD_; ^#^ indicates statistical comparison between Spike_RBD_ + adjuvant vs. VHH_MHCII_-Spike_RBD_ + adjuvant cohorts, whereas * indicates statistical comparison between adjuvant only vs. VHH_MHCII_-Spike_RBD_ + adjuvant cohorts; ^#,^*P < 0.05, ^##,^**P < 0.01, ^###,^***P < 0.001, unpaired *t* test with Holm–Sidak adjustment. (*D*) IFNγ, IL-6, IL-2, and TNFα levels were measured 3 d after stimulating splenocytes with the indicated Spike_RBD_ peptide; ^&^^,^^#^, and * indicate statistical comparisons between adjuvant only vs. Spike_RBD_ + adjuvant cohorts, Spike_RBD_ + adjuvant vs. VHH_MHCII_-Spike_RBD_ + adjuvant cohorts, and adjuvant only vs. VHH_MHCII_-Spike_RBD_ + adjuvant cohorts, respectively; ^&,#,^*P < 0.05, ^&&,##,^**P < 0.01, ^###,^***P < 0.001, unpaired *t* test with Holm–Sidak adjustment. (*E* and *F*) Flow cytometry analyses of splenocytes after incubation for 6 h in the presence of pooled peptides (42 and 47 to 50) and monensin. All data are presented as means ± SEM; *P < 0.05, **P < 0.01, ***P < 0.001, unpaired *t* test with Holm–Sidak adjustment.

Because cellular immunity, particularly that exerted by T cells, is important for protection against and memory of SARS-CoV-2 infection, we next examined the T cell response in animals immunized with a single dose of adjuvanted Spike_RBD_ or VHH_MHCII_-Spike_RBD_ (Fig. 2*A*) (5). Splenocytes harvested 7 d postimmunization were stimulated with a library of Spike_RBD_ 15-mer peptides with 11-residue overlap and evaluated by an ELISpot assay (Fig. 2*B* and Table 1). While mouse class I MHC products prefer peptides in the eight to nine residue range, we chose to use synthetic 15-mers, as they are known to contain shorter peptides that serve as class I MHC ligands or can be processed to yield proper class I MHC ligands (22, 23). The use of longer peptides would enable identification of both class I and class II restricted epitopes. We observed robust production of IFNγ in splenocytes from mice immunized with VHH_MHCII_-Spike_RBD_ and, to a lesser extent, from mice immunized with Spike_RBD_ (Fig. 2*C*). IFNγ production was highest when splenocytes were stimulated with peptides 42, 47, 48, 49, and 50, indicative of at least two unique stimulatory regions. Peptide 42 contains residue E484, which is frequently mutated to K or Q in SARS-CoV-2 variants, and S494, which is sometimes mutated to P in the B.1.1.7 lineage. Peptides 47 to 50, which correspond to spike residues 503 to 529, are conserved among all Centers for Disease Control–designated variants (17). Indeed, coculture of splenocytes from VHH_MHCII_-Spike_RBD_–immunized mice with Spike_RBD_ peptides 42 and 47 to 50 resulted in secretion not only of high levels of IFNγ but also high levels of the proinflammatory cytokines IL-6 and TNFα. These levels are higher than those induced by immunization with Spike_RBD_ and indicate proliferation of memory CD4 and CD8 T cells (Fig. 2*D*) (24, 25). We did not observe high levels of IL-2 secretion. Cellular immunity elicited by immunization with VHH_MHCII_-Spike_RBD_ is strong and may persist upon infection with circulating variants.

**Table 1.** Peptide sequences for splenocyte stimulation experiments

No.	Peptide sequence	No.	Peptide sequence	No.	Peptide sequence
1	RV_320_QPTESIVRFP_330_NIT	19	CFTNVYADSF_400_VIRGD	37	PFERDIST_470_EIYQAGS
2	TESIVRFP_330_NITNLCP	20	VYADSF_400_VIRGDEVRQ	38	DIST_470_EIYQAGSTPC_480_N
3	VRFP_330_NITNLCPFGE_340_V	21	SF_400_VIRGDEVRQI_410_APG	39	EIYQAGSTPC_480_NGVEG
4	NITNLCPFGE_340_VFNAT	22	RGDEVRQI_410_APGQTGK	40	AGSTPC_480_NGVEGFNCY
5	LCPFGE_340_VFNATRFAS	23	VRQI_410_APGQTGKIAD_420_Y	41	PC_480_NGVEGFNCYF_490_PLQ
6	GE_340_VFNATRFASV_350_YAW	24	APGQTGKIAD_420_YNYKL	42	VEGFNCYF_490_PLQSYGF
7	NATRFASV_350_YAWNRKR	25	TGKIAD_420_YNYKLPDDF	43	NCYF_490_PLQSYGFQPT_500_N
8	FASV_350_YAWNRKRISN_360_C	26	AD_420_YNYKLPDDFT_430_GCV	44	PLQSYGFQPT_500_NGVGY
9	YAWNRKRISN_360_CVADY	27	YKLPDDFT_430_GCVIAWN	45	YGFQPT_500_NGVGYQPYR
10	RKRISN_360_CVADYSVLY	28	DDFT_430_GCVIAWNSNN_440_L	46	PT_500_NGVGYQPYRV_510_VVL
11	SN_360_CVADYSVLYN_370_SAS	29	GCVIAWNSNN_440_LDSKV	47	VGYQPYRV_510_VVLSFEL
12	ADYSVLYN_370_SASFSTF	30	AWNSNN_440_LDSKVGGNY	48	PYRV_510_VVLSFELLHA_520_P
13	VLYN_370_SASFSTFKCY_380_G	31	NN_440_LDSKVGGNYN_450_YLY	49	VVLSFELLHA_520_PATVC
14	SASFSTFKCY_380_GVSPT	32	SKVGGNYN_450_YLYRLFR	50	FELLHA_520_PATVCGPKK
15	STFKCY_380_GVSPTKLND	33	GNYN_450_YLYRLFRKSN_460_L	51	HA_520_PATVCGPKKS_530_TNL
16	CY_380_GVSPTKLNDL_390_CFT	34	YLYRLFRKSN_460_LKPFE	52	TVCGPKKS_530_TNLVKNK
17	SPTKLNDL_390_CFTNVYA	35	LFRKSN_460_LKPFERDIS	53	PKKS_530_TNLVKNKCVN_541_F
18	LNDL_390_CFTNVYADSF_400_V	36	SN_460_LKPFERDIST_470_EIY		

To distinguish between CD4 and CD8 T cells as the dominant cytokine-producing cell type, we performed intracellular cytokine staining. A significant proportion of the inflammatory cytokines are derived from the CD8 T cell compartment (Fig. 2 *E* and *F* and *SI Appendix*, Fig. S5). Recombinant VHH_MHCII_-antigen fusion proteins are therefore subject to cross-presentation and can induce an efficacious CD8 T cell response against Spike_RBD_. The presence of a strong humoral immune response that includes class switching likewise implies the contribution by CD4 T cells (Fig. 2*F* and *SI Appendix*, Fig. S5).

### VHH_MHCII_-Spike_RBD_ Elicits a Strong Humoral Response Regardless of the Route of Administration, Storage Temperature, Formulation, and Age of the Mice.

**Fig. 3.**
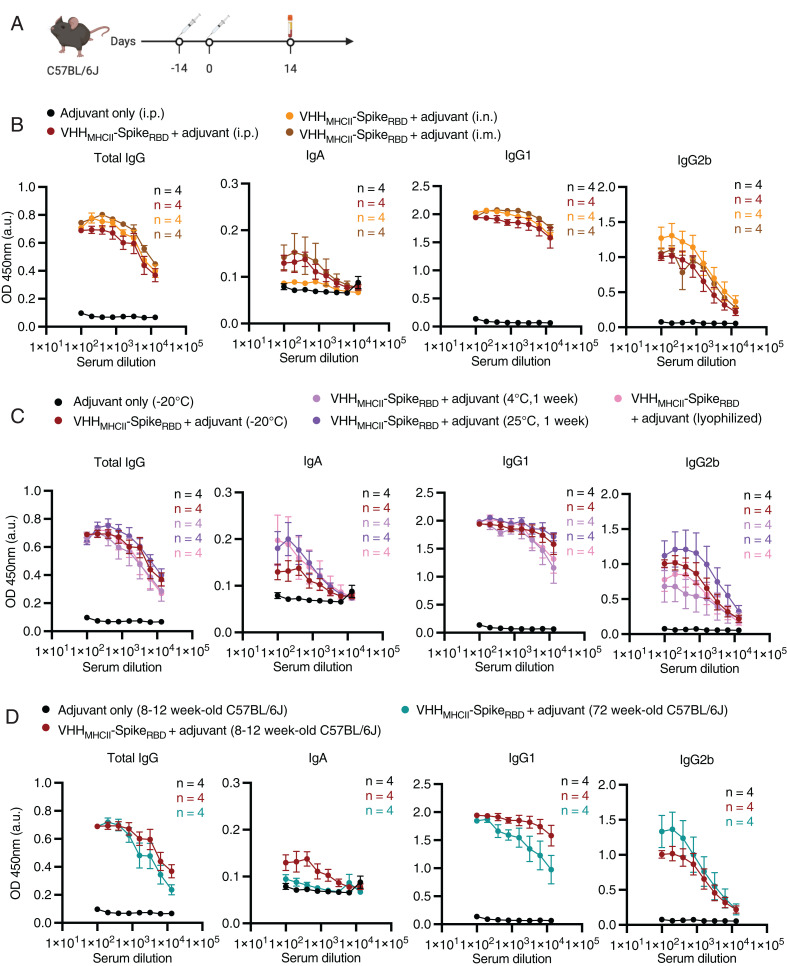
VHH_MHCII_-Spike_RBD_ elicits a strong humoral response, regardless of route of administration, storage temperature of the vaccine, lyophilization of the vaccine, and age. (*A*) C57BL/6J mice were immunized with two adjuvanted doses (days 0 and 14) of VHH_MHCII_-Spike_RBD_ under various conditions highlighted in *B*–*D*, and serum was collected 14 d postboost. (*B*) IgG, IgA, IgG1, and IgG2b levels were measured against recombinant Spike_RBD_ following immunization with VHH_MHCII_-Spike_RBD_ by i.p., i.n., or i.m. administration. Control mice were immunized by the i.p. route with two doses of adjuvant alone. (*C*) IgG, IgA, IgG1, and IgG2b levels were measured against recombinant Spike_RBD_ following immunization with two doses of adjuvant only, adjuvanted VHH_MHCII_-Spike_RBD_ incubated at either −20 °C, 4 °C, or 25 °C for 1 wk, or with lyophilized and resuspended, adjuvanted VHH_MHCII_-Spike_RBD_. (*D*) IgG, IgA, IgG1, and IgG2b levels were measured against recombinant Spike_RBD_ following immunization of 8- to 12 wk-old mice with two doses of adjuvant only or adjuvanted VHH_MHCII_-Spike_RBD_, or 72-wk-old mice with two doses of adjuvanted VHH_MHCII_-Spike_RBD_. For *B*–*D*, *n* = 4 for all conditions, and curves are plotted as means of each condition. OD, optical density.

We next compared different routes of administration, including i.p., intramuscular (i.m.), and intranasal (i.n.) routes, to elicit an immune response. Mice were primed with adjuvant only (i.p.) or received adjuvanted VHH_MHCII_-Spike_RBD_ (i.p., i.m., or i.n.) and boosted 14 d later with a dose of the corresponding vaccine and route of administration (Fig. 3*A* and *SI Appendix*, Fig. S6*A*). Mean total Spike_RBD_-specific IgG in the blood, as well as mean IgM, IgG1, and IgG2b levels, were comparable across the three different routes of administration, although i.p. and i.m. administration led to an increase in mean IgA levels (Fig. 3*B* and *SI Appendix*, Fig. S6*A*). While i.n. administration did not induce significant levels of IgA in the blood, it remains to be determined whether these animals produced significant levels of IgA in the respiratory tract.

Because the SARS-CoV-2 vaccines in use require specific formulations and cold storage conditions, we determined whether recombinant VHH_MHCII_-Spike_RBD_ protein would retain its efficacy at inducing an antibody response upon storage at ambient temperature or lyophilization. We immunized mice with two doses of adjuvant only, adjuvanted VHH_MHCII_-Spike_RBD_ stored at −20 °C, 4 °C, or 25 °C for 1 wk, or lyophilized and resuspended VHH_MHCII_-Spike_RBD_ (Fig. 3*A* and *SI Appendix*, Fig. S6*B*). Mean total serum IgG, IgA, IgG1, IgG2b, and IgM levels between the different conditions were comparable, indicating that neither temperature of storage nor storage in liquid form is required for the vaccine to retain its efficacy (Fig. 3*C*).

Because the ability to produce a robust and durable immune response can decrease with age, we also determined whether immunization of aged mice with VHH_MHCII_-Spike_RBD_ would elicit a strong immune response (26). We immunized 8- to 12-wk-old mice with two doses of adjuvant only or adjuvanted VHH_MHCII_-Spike_RBD_ and 72-wk-old [equivalent to humans aged 56 y to 69 y (27)] with two doses of VHH_MHCII_-Spike_RBD_ (Fig. 3*A* and *SI Appendix*, Fig. S6*C*). While switching to some immunoglobulin subclasses (IgA and IgG1) decreased in aged mice, consistent with diminished T cell help, mean total IgG levels in the blood were unchanged when compared to 8- to 12-wk-old mice (Fig. 3*D*). This may be due, in part, to high levels of IgG2b in aged mice and slightly higher levels of IgM (*SI Appendix*, Fig. S6*C*).

### Humanized VHH_hMHCII_-Spike_RBD_ Elicits Both Humoral and Cellular Immunity in a Transgenic Mouse Model.

**Fig. 4.**
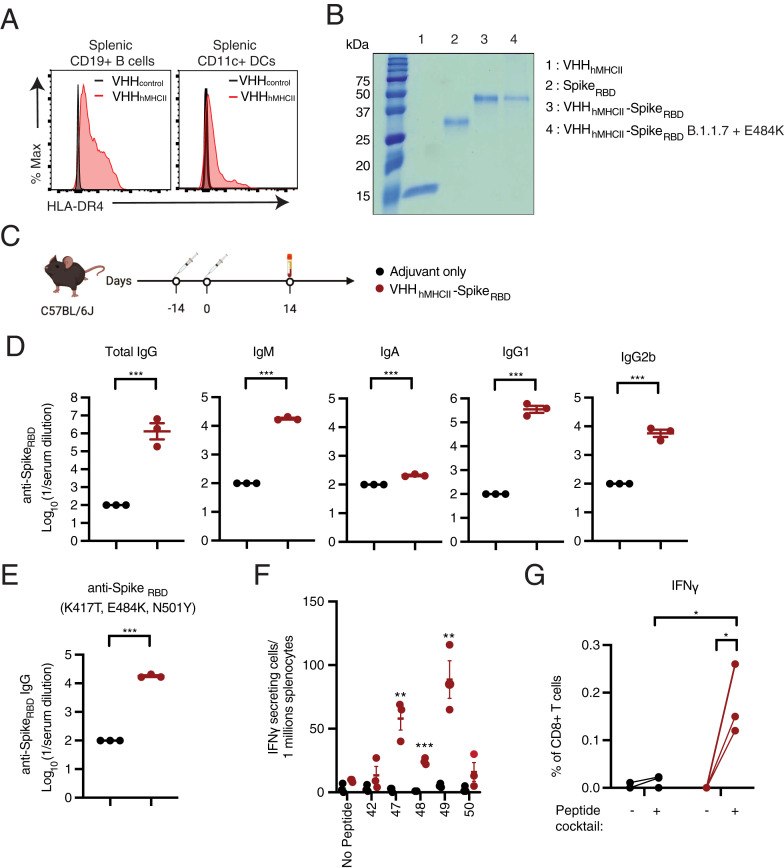
Anti-human VHH_hMHCII_ conjugated to Spike_RBD_ generates humoral and cellular immunity in a transgenic mouse model. (*A*) Flow cytometry analyses of splenocytes from DR4-IE–transgenic C57BL/6 *IAb*^null^ mice to detect cell surface binding of VHH_hMHCII_. (*B*) Instant Blue–stained SDS/PAGE gel showing recombinant, purified VHH_hMHCII_, Spike_RBD_, VHH_hMHCII_-Spike_RBD_ (Wuhan Hu-1), and VHH_hMHCII_-Spike_RBD_ (B.1.1.7+E484K). (*C*) DR4-IE–transgenic C57BL/6 *IAb*^null^ mice were immunized with two doses (days 0 and 14) of adjuvant only or adjuvanted VHH_hMHCII_-Spike_RBD_ (Wuhan Hu-1); *n* = 3 per condition. Serum samples were collected 14 d postboost. (*D*) IgG, IgM, IgA, IgG1, and IgG2b levels were measured by ELISA against recombinant Spike_RBD_. ELISA data were summarized as endpoint titers and presented as means ± SEM. (*E*) Total IgG levels were measured from immunized mice by ELISA against recombinant Spike_RBD_ containing the mutations K417T, E484K, and N501Y. ELISA data were summarized as endpoint titers and presented as means ± SEM. (*F*) ELISpot assay measuring IFNγ-secreting cells in splenocytes of DR4-IE–transgenic C57BL/6 *IAb*^null^ mice (*n* = 3 per condition) immunized with one dose of adjuvant only or adjuvanted VHH_hMHCII_-Spike_RBD_. Splenocytes were harvested at day 7 post immunization. (*G*) Flow cytometry analyses of splenocytes after incubation for 6 h in the presence of pooled peptides (42 and 47 to 50) and monensin. All data are presented as means ± SEM; *P < 0.05, **P < 0.01, ***P < 0.001, unpaired *t* test with Holm–Sidak adjustment.

Because the anti-MHC class II nanobody used for these experiments recognizes murine antigens independent of haplotype, we also generated a version of the vaccine that could be applied in a clinical setting. We used HLA-DR4-IE–transgenic C57BL/6 *IAb*^null^ mice, which lack wild-type murine MHC class II products and instead express transgenic hybrid MHC class II molecules composed of the peptide-binding portion of human HLA-DR4 and the membrane-proximal domains of mouse I-E (DR4-IE). We used a previously characterized nanobody (VHH_hMHCII_) that recognizes nearly all allelic variants of human class II MHC molecules (HLA-DR specific, with the exception of HLA-DR-03*01) (VHH_hMHCII_-Spike_RBD_) (28). Flow cytometry on splenocytes from HLA-DR4-IE–transgenic C57BL/6 *IAb*^null^ mice confirmed that VHH_hMHCII_ recognizes these hybrid DR4-IE molecules (Fig. 4*A*). We then generated a genetic fusion between VHH_hMHCII_ and either the Wuhan Hu-1 or B.1.1.7+E484K SARS-CoV-2 Spike_RBD_, as an illustration of rapid and straightforward vaccine adjustment in anticipation of emerging mutations in the RBD. Both constructs expressed well in mammalian cells. A 200-mL culture of Expi293 cell supernatant yielded 15 and 12.5 mg of VHH_hMHCII_-Spike_RBD_ (Wuhan Hu-1) and VHH_hMHCII_-Spike_RBD_ (B.1.1.7+E484K), respectively (Fig. 4*B*).

To examine the immunogenicity of this vaccine, we immunized HLA-DR4-IE–transgenic C57BL/6 *IAb*^null^ mice by i.p. administration with two doses of adjuvant only or 20 μg of adjuvanted VHH_hMHCII_-Spike_RBD_ (Wuhan Hu-1 RBD) (Fig. 4*C* and *SI Appendix*, Fig. S7*A*). Immunization with VHH_hMHCII_-Spike_RBD_ elicited high-titer anti-Spike_RBD_ antibodies, reaching mean endpoint titers of 1/1,296,724 (total IgG), 1/17,853 (IgM), 1/208 (IgA), 1/349,669 (IgG1), and 1/5,707 (IgG2b) (Fig. 4*D* and *SI Appendix*, Fig. S7 *B* and *C*). Total serum IgG retained the ability to bind mutant, recombinant Spike_RBD_ with the K417T, E484K, and N501Y mutations found in the P.1 variant at mean endpoint titers of 1/17,853 (Fig. 4*E* and *SI Appendix*, Fig. S7*D*). We also identified three peptides, 47, 48, and 49, that elicited strong IFNγ responses in splenocytes after immunization with a single dose (Fig. 4*F*). Not surprisingly, CD8 T cells are the dominant cell type implicated in IFNγ production upon peptide stimulation (Fig. 4*G*), as the HLA-DR4-IE–transgenic C57BL/6 *IAb*^null^ mice share the H-2^b^ encoded class I MHC molecules.

## Discussion

Curtailing the SARS-CoV-2 pandemic will require rapid and widespread distribution of effective vaccines. Here we report the development of VHH_MHCII_-Spike_RBD_, a purely protein-based SARS-CoV-2 vaccine that specifically targets APCs. This preparation is easy to both produce and store. Immunization of mice with two doses of VHH_MHCII_-Spike_RBD_ elicited high-titer binding and neutralizing antibodies against SARS-CoV-2 and several of its circulating variants, including B.1.1.7, P.1, and B.1.351. Strong immune responses were evoked in both young and aged mice, largely independent of the route of administration of the vaccine. A single dose was sufficient to induce cellular immunity to conserved regions of the RBD, as evident from cytokine production by CD8 T cells. The vaccine maintained its potency regardless of storage conditions, including ambient temperature and lyophilization. Humoral and cellular immune responses were both more consistent and potent in mice immunized with VHH_MHCII_-Spike_RBD_ compared to immunization with the Spike_RBD_. A version of this vaccine suitable for clinical translation elicits robust immunity in a humanized mouse model. This approach would therefore complement ongoing active and passive immunization strategies.

Most currently used vaccines are difficult to manufacture and/or require specialized storage conditions. Vaccines with enhanced stability that tolerate lyophilization, such as the protein-based vaccine reported here or a different, nanoparticle-based vaccine, allow stockpiling at ambient temperature (29). This is an important attribute for distribution in countries where access to cold storage and/or effective transportation is a challenge. A unique feature of this vaccine approach is its ability to deliver antigen directly to MHC class II^+^ cells. Humoral and cellular immunity directed against the Spike_RBD_ develops within a week of the first dose, unlike mRNA-based vaccines and nontargeting protein-based vaccines, which usually take longer (30).

In conclusion, we report a purely protein-based vaccine preparation that is unique, in that it directly targets professional APCs. The robust immunity afforded by this vaccine, combined with its ease of manufacture and stability, indicates the potential for a rapidly adjustable vaccine. Vaccination of mice with the VHH_MHCII_-Spike_RBD_ adduct elicits CD4 and CD8 T cell as well as B cell responses, resulting in the formation of antibodies that neutralize not only recombinant VSV expressing the SARS-CoV-2 spike but also a SARS-CoV2 isolate. Further studies in small animal models and nonhuman primates are needed to establish whether immunity elicited by VHH_MHCII_-Spike_RBD_ protects against a SARS-CoV-2 challenge and to establish breadth of coverage. We suggest that this approach merits consideration for use in a clinical setting as a complement to ongoing active and passive immunization strategies.

## Materials and Methods

### Cells and Antibodies.

Expi293F cells were maintained in humidified, shaking incubators at 37 °C, 8% CO_2_ in Expi293 Expression Media (ThermoFisher Scientific). BSRT7/5, Vero-CCL81, Vero E6-TMPRSS2, and Vero-hACE2-TMPRSS2 cells were maintained in humidified incubators at 34 °C or 37 °C and 5% CO_2_ in Dulbecco’s modified Eagle’s medium (DMEM) (Corning) supplemented with glucose, L-glutamine, sodium pyruvate, and 10% fetal bovine serum (FBS). Vero E6, Vero E6-TMPRSS2, and Vero-hACE2-TMPRSS2 cells were described previously (31). Vero E6 cells (CRL-1586, ATCC) were grown in DMEM (Gibco) supplemented with 5% fetal calf serum (Sigma), 100 U/mL penicillin (Gibco), 100 µg/mL streptomycin (Gibco), and 2 mM L-glutamine (Lonza). All commercially available antibodies utilized in this study are indicated in Table 2.

**Table 2.** Commercial antibodies utilized in this study

Target	Color	Clone	Manufacturer	Catalog number
CD45	BV605	30-F11	Biolegend	103139
CD3	PerCPCy5.5	17A2	Biolegend	100218
CD4	PeCy7	RM4-5	Biolegend	100528
CD8α	FITC	53–6.7	Biolegend	100706
TNFα	APC	MP6-XT22	eBioscience	17-7321-82
IL2	BV421	JES6-5H4	Biolegend	503826
IFNγ	PE	XMG1.2	eBioscience	12-7311-82
Fc block (CD16/CD32)	Not Applicable	93	Biolegend	101302

### Viruses.

Generation of replication-competent VSV-SARS-CoV-2 (Wuhan Hu-1, GenBank MN908947.3) has been described (19). Additional VSV recombinants expressing eGFP and SARS-CoV-2 spike variants were prepared as follows. Briefly, spike genes of Wuhan Hu-1+D614G, B.1.1.7 (GenBank OU117158.1), B.1.351 (GenBank MZ212516.1), and P.1 (GISAID EPI_ISL_804823) were truncated to remove the C-terminal 63 nucleotides and were then cloned into an infectious molecular clone (complementary DNA [cDNA]) of VSV-eGFP in place of the native VSV G gene. To rescue the recombinant VSVs, BSRT7/5 cells were infected with vaccinia virus encoding the bacteriophage T7 RNA polymerase (vTF7-3) and subsequently transfected with T7-driven support plasmids encoding VSV N, P, L, and G, as well as the infectious molecular cDNAs. Cell supernatants were harvested ∼72 h postinfection, clarified by centrifugation (5 min and 1,000 × *g*), and filtered through a 0.22-μm filter. Rescue supernatants were plaque purified on Vero-CCL81, Vero E6-TMPRSS2, or Vero-hACE2-TMPRSS2 cells in the presence of 25 μg/mL cytosine arabinoside (AraC). Plaques in agarose plugs were grown on Vero-CCL81, Vero E6-TMPRSS2, or Vero-hACE2-TMPRSS2 cells also in the presence of 25 μg/mL AraC to generate P1 stocks. Working stocks were generated on Vero-CCL81 or Vero E6-TMPRSS2 cells at 34 °C. SARS-CoV-2 strain NL/2020 (EVAg, Ref-SKU 010V-03903) was propagated and titrated on Vero E6 cells using the tissue culture infective dose 50 (TCID50) endpoint dilution method.

### Design, Expression, and Purification of Recombinant VHHs and VHH Fusions.

Sequences encoding the Spike_RBD_ and VHH_MHCII_-Spike_RBD_ were synthesized (Integrated DNA Technologies) as double-stranded DNA. Inserts were assembled in the pVRC vector (a gift from Stephen Harrison, Harvard Medical School). Constructs were transfected into Expi293F cells (Thermo Fischer Scientific) using Polyethyleneimine “Max” (Polysciences). Cell cultures were maintained in Expi293 Media (Thermo Fischer Scientific) at 37 °C for 4 d following transfection. Proteins were harvested by centrifugation at 5,000 × g for 30 min at 4 °C, followed by affinity chromatography with HisPur Ni-NTA Resin (Thermo Fischer Scientific) and size exclusion chromatography with a Hi-Load 16/600 S75 column (Cytivia). Protein samples (2 μg each) were prepared by boiling for 5 min in sample buffer containing 1% (wt/vol) SDS and 1% (vol/vol) BME. Samples were analyzed on 10% or 15% SDS/PAGE. Gels were stained with Instant Blue (Abcam) and destained with ddH_2_O.

### Mouse Models.

All animals were housed in the animal facility of Boston Children’s Hospital (BCH) and were maintained according to protocols approved by the BCH Committee on Animal Care. C57BL/6J (CD45.2+) and BALB/c mice were either purchased from the Jackson Laboratory or bred in house. DR4-IE transgenic mice were purchased from Taconic. Only female mice aged 8 to 12 wk were used in this study unless indicated otherwise.

### ELISA.

Serum samples were collected on the indicated days and stored in BD Vacutainers; 96-well plates were coated with 2 μg/mL of either recombinant Spike_RBD_ or Spike_RBD_ (K417T, E484K, N501Y) proteins in phosphate-buffered saline (PBS) overnight at 4 °C and incubated in blocking buffer (0.05% Tween20 + 2% bovine serum albumin in PBS). Plates were incubated with diluted serum samples for 2 h at room temperature. Plates were then washed four times with PBS and incubated with goat anti-mouse IgG-HRP, anti-mouse IgM-HRP, anti-mouse IgG1-HRP, or IgG2b-HRP (SouthernBiotech) at 1:10,000 or with IgA-HRP at 1:2,000 in blocking buffer for 1 h. Plates were developed with 3,3',5,5'-tetramethylbenzidine liquid substrate reagent (Sigma). The reaction was stopped with 1 N HCl, and absorbance was read at 450 nm.

### Recombinant VSV Neutralization Assays.

Serum samples were heat inactivated at 56 °C for 30 min. Neutralization assays were performed similarly to what has been described (19, 20). Briefly, threefold serial dilutions of sera, starting with a 1:20 dilution, were performed in 384-well plates and were incubated with 10^6^ plaque-forming units (pfu) of VSV-SARS-CoV-2 expressing eGFP and the Spike of Wuhan Hu-1+D614G, B.1.1.7, P.1, or B.1.351 for 1 h at 37 °C. Vero E6 cells then were added to the human serum–virus complexes in 384-well plates at 3 × 10^3^ cells per well and incubated at 37 °C for 16 h. Cells were fixed at room temperature in 4% formaldehyde and then rinsed with PBS. Cells were stained at room temperature with NucRed Live 647 (Invitrogen) for 30 min. Images were acquired using an InCell 6500 confocal imager (Cytiva) to visualize nuclei and infected cells (4× objective, 1 field per well). Images were segmented using InCarta (Cytiva). Infected cells were identified by comparing them to the uninfected threshold in Spotfire (Tibco). Cells were gated based on nuclear parameters.

Additional VSV neutralization assays were performed similarly to what has been described (19). Briefly, serial dilutions of serum samples were incubated with ∼10^2^ pfu of VSV-SARS-CoV-2 for 1 h at 37 °C. Antibody–virus complexes were then added to Vero CCL-81 cells in black 96-well plates for 7.5 h at 37 °C. Cells were then fixed in 2% (vol/vol) formaldehyde (Millipore Sigma) containing 10 mg/mL Hoechst 33342 nuclear stain (Invitrogen) for 45 min at room temperature. Following incubation, nuclear stain and fixative were removed and replaced with PBS. Images were acquired with the InCell 2000 Analyzer (GE Healthcare) automated microscope in both the DAPI and fluorescein isothiocyanate (FITC) channels to visualize nuclei and infected cells (i.e., eGFP-positive cells), respectively (4× objective, four nonoverlapping fields per well, covering the entire well). Images were analyzed using the InCell Investigator Developer Toolbox v1.9 (GE Healthcare). Nuclei and GFP-positive cells were identified and segmented from their respective images using the Object Segmentation function, which uses a variation of the top-hat approach for initial segmentation followed by a binarization operation to define the target sets. Target linking was then performed to determine the total number of GFP-positive nuclei per well. Data were processed using Prism software (GraphPad Prism 9.0).

### SARS-CoV-2 Neutralization Assay.

Neutralization assays using SARS-CoV-2 were performed similarly as described (32). Briefly, Vero E6 cells were seeded 1 d prior to infection at 12,000 cells per well in a 96-well tissue culture plate. Serum samples were heat inactivated (56 °C for 30 min) and serially diluted (dilution range of 1:10 to 1:1,280) in 60 μL of DMEM supplemented with penicillin, streptomycin, L-glutamine, and 2% fetal calf serum (FCS). The diluted sera were mixed with equal volumes of 120 TCID50/60 µL of SARS-CoV-2 strain NL/2020 and incubated for 1 h at 37 °C. The virus–serum mixtures were then applied onto the previously seeded Vero E6 cells and incubated at 37 °C for 2.5 d. Cells infected with 120 TCID50/60 µL of SARS-CoV-2 strain/NL/2020 were used as a positive control. As a neutralization control, we added mAb 47D11 group, which is a human monoclonal antibody that can neutralize SARS-CoV-2. In brief, mAB 47D11 (3.14 mg/mL) was prediluted 1:100 (to 31.4 ug/mL) in DMEM (+2% FCS, PenStrep, glutamine) followed by the serial dilution indicated in the figures. This mAb 47D11 was kindly provided by B. J. Bosch, Utrecht University, Utrecht, The Netherlands. At 2.5 d postinfection, supernatant was harvested and heat inactivated at 75 °C for 30 min. Real-time RT-PCR of the SARS-CoV-2 E gene was performed on the supernatant samples as described previously (33). All work with live SARS-CoV-2 was performed in a biosafety level 3 facility in the University Medical Center Utrecht.

### Peptide Synthesis.

The RBD of the spike protein was divided into 53 overlapping 15-mer peptides with an overlap of 11 amino acids (Table 1). The peptides were obtained from GenScript as lyophilized powder. The peptides were resuspended at a concentration of 4 mg/mL in water (with up to 5% dimethyl sulfoxide) and stored at −20 °C.

### ELISpot Assay.

IFNγ ELISpot assays (BD ELISPOT Mouse IFNγ ELISPOT Set, BD Biosciences) were performed according to the manufacturer’s instructions. Briefly, 96-well ELISpot plates were coated with an IFNγ capture antibody (BD Biosciences, 51-2525K) in PBS overnight at 4 °C. Plates were then blocked with complete growth medium for 2 h at room temperature. Wells were then emptied, and 1 × 10^6^ splenocytes from immunized mice were added to the plates. Cells were arrayed in the presence or absence of 15-mer peptides with 11-residue overlaps derived from the SARS-CoV-2 Spike_RBD_ (10 μg/mL) in 200 μL of complete medium and incubated overnight at 37 °C. Plates were then washed and incubated with a biotinylated IFNγ detection antibody (BD Biosciences, 51-1818KZ) for 2 h at room temperature and incubated with streptavidin-horseradish peroxidase (BD Biosciences) for 1 h at room temperature. Plates were developed with 3-amino-9-ethyl-carbazole substrate (BD ELISPOT AEC Substrate Set) for 5 min to 30 min and dried overnight. Spots were enumerated using the KS ELISpot analysis system (Immunospot).

### Cytokine Secretion Assay.

Three days poststimulation with Spike_RBD_ 15-mer peptides, cell supernatants were collected and used for ELISA to measure IFNγ, IL-6, IL-2, and TNFα production. IFNγ, IL-2, IL-6, and TNFα were measured using the BD OptEIA Mouse IFN-γ ELISA Set (BD Biosciences, 555138), BD OptEIA Mouse IL-6 ELISA Set (BD Biosciences, 555240), BD OptEIA Mouse IL-2 ELISA Set (BD Biosciences, 555148), and Mouse TNFα Uncoated ELISA kit (Invitrogen, 88-7324-22) per manufacturer's protocol.

### Flow Cytometry Analyses.

Cells harvested from excised spleens were dispersed into RPMI 1640 (Gibco) through a 40-μm cell strainer using the back of a 1-mL syringe plunger. Cell mixtures were subjected to hypotonic lysis (NH_4_Cl) to remove red blood cells, washed twice in flow cytometry buffer (2 mM (ethylenedinitrilo)tetraacetic acid and 1% FBS in PBS) and resuspended in flow cytometry buffer containing the corresponding fluorescent dye-conjugated antibodies. Staining steps were carried out at 1:100 dilutions in the presence of Fc block (Biolegend) for 30 min at 4 °C in the dark. Samples were washed twice with fluorescence-activated cell sorter (FACS) buffer before further analysis. All flow data were acquired on a FACS Fortessa flow cytometer (BD Biosciences) and analyzed using FlowJo software (Tree Star).

### Lyophilization of Vaccine Preparations.

VHH_MHCII_-Spike_RBD_ combined with adjuvant in PBS were flash frozen in a 2-mL Eppendorf tube. The tube was opened, placed in a 300-mL flask, and lyophilized overnight using a Freezone 4.5 Plus Freeze Dryer (Labconco). After overnight lyophilization, the resulting powder was left at room temperature for 1 wk before immunization.

### Statistical Methods.

All data represent at least two independent experiments. All statistical analyses were performed using Prism 6. Statistical methods used are indicated in the corresponding legend of each figure.

## Data Availability

All data that support the findings in this publication are included in the article and *SI Appendix*.
